# What kind of children do kindergarten teachers prefer? —Typology of the preferred types of children

**DOI:** 10.3389/fpsyg.2024.1291350

**Published:** 2024-02-21

**Authors:** Song Shi, Huayuan Zhang, Zizai Zhang, Jiahui Sun, Xin Qi, Shunian Li

**Affiliations:** ^1^School of Education Science, Nantong University, Nantong, Jiangsu, China; ^2^Fuyang Preschool Teachers College, Fuyang, Anhui, China; ^3^College of Child Development and Education, Zhejiang Normal University, Hangzhou, China; ^4^College of Education, Jeonju University, Jeoju, Republic of Korea; ^5^Department of Education, The Catholic University of Korea, Bucheon, Republic of Korea; ^6^School of International Education, Tongren Preschool Education College, Tongren, China

**Keywords:** kindergarten teachers, child, preference, teacher’s pet phenomenon, children teachers prefer

## Abstract

The teacher’s pets are a common occurrence in the field of education. To investigate the preferences teachers exhibit toward certain children, the study focused on kindergarten teachers and employed a mixed research methodology. Initially, qualitative interviews were conducted with 15 kindergarten teachers to identify specific criteria influencing teacher preferences. Subsequently, A comprehensive model of teacher’s pets was developed through a questionnaire survey involving 463 participants. This model encapsulated 32 distinct indicators, categorized into 7 types: children with good appearance (GA), exceptional abilities (OA), commendable conduct (GC), proactive and enthusiastic demeanor (PE), compliant and carefree nature (OC), children from vulnerable groups (VC), and those influenced by their parents (PI). The resulting model demonstrated a sound structure. Not only did it validate existing findings, but it also expanded upon the identified types of teacher’s pets. An analysis based on game theory revealed the weighted combinations, highlighting the top three types of teacher’s pets: children influenced by parental factors (24.3%), proactive and enthusiastic individuals (15.7%), and obedient, carefree children (14.8%), respectively. Conversely, the representation of vulnerable-concerned children (11.1%) was the lowest among the identified types.

## 1 Introduction

Equity in education remains an enduring concern, advocating for equal treatment among all educators, achieving this goal persists as a challenge (Anne Winter, 2018; Psaki et al., 2018). Inequities within education manifest not solely in national resource allocation but also in the dynamics between teachers and students within classroom settings (Vahidnia et al., 2019). The Study predominantly focuses on fairness, centering on vulnerable groups: girls, children from ethnic minorities, individuals with disabilities, students from impoverished families, and those encountering academic disadvantages (Klopfenstein, 2004; Belfiore et al., 2005; Subrahmanian, 2005; Rioux and Pinto, 2010; Vincent and Shobha, 2020). Within the teacher-student relationship, the Teacher’s Pet Phenomenon (TPP) delineates a unique emotional bond between a teacher and a specific student or group, often characterized by favoritism where the favored student is labeled the “teacher’s pet” (Babad, 2009). This favoritism results in preferential treatment for teacher’s pets due to the teachers’ preferences, contradicting the core principles of educational fairness (Tal and Babad, 1989; Tal and Babad, 1990; Luttrell, 1993; Trusz, 2017). Teacher favoritism may have positive or negative effects on students (Brey and Pauker, 2019). Previous studies have predominantly explored the teachers’ pet phenomenon from the students’ perspective, few scholars have delved into the phenomenon from the viewpoint of teachers. However, teachers and students exhibit distinct attitudes toward the teachers’ pet phenomenon, with notable differences in their responses to this occurrence (Tal and Babad, 1989). Hence, there is a need to enhance study on this phenomenon from the teachers’ perspective. Before this study, no scholars had undertaken specialized empirical study on the categorization of teachers’ pets at any educational stage. Thus, this study focused on kindergarten teachers as the subjects of study, examining the students whom teachers tend to favor. It aimed to systematically categorize and understand the various types of kindergarten teachers’ pets from the viewpoint of these educators.

### 1.1 Teacher’s pet phenomenon

Silberman (1969) stands as a pioneering figure in delving into the phenomenon of teachers’ pets. He delineated four emotional stances that teachers adopt toward particular students: attachment, concern, indifference, and rejection. Silberman posited that “attachment” signifies an emotional bond between teachers and students who bring them satisfaction, often leading these students to become teachers’ pets. This attachment encapsulates a warm connection, stemming from the joy that students bring to teachers’ professional lives. Building upon this foundation, Babad and his colleagues conducted an extensive exploration of teachers’ pets through empirical studies in the 1990s. Tal and Babad (1989) and Babad (1995) classified classrooms into three categories based on the number of favored students: those devoid of pets, those with a single exclusive pet student, and those with multiple favored students. The prevalence of teachers having pet students is a widespread occurrence. Surveying 80 fifth-grade classrooms in Israel revealed that 80% of the classrooms exhibited the pet phenomenon (26% exclusively favored a single student, while 54% had multiple favored students) (Tal and Babad, 1990). Moreover, a staggering 90% of respondents acknowledged experiencing teachers’ pets in their classrooms (Tal and Babad, 1989). Subsequent studies corroborated the ubiquity of teachers’ pets across all academic levels (Babad et al., 1989; Somersalo et al., 2002; Chiu et al., 2013; Vahidnia et al., 2021).

Tal and Babad (1989) discovered that both students and teachers view the concept of teachers’ pets in a negative light, with students exhibiting a more pronounced negative reaction than teachers. Students respond with frustration toward teachers favoring specific students, focusing on perceived negatives like favoritism, preference, and unfairness (Luttrell, 1993; Babad, 1995). Those not favored often experience jealousy witnessing preferential treatment toward others, feeling neglected and unfairly treated by teachers (Martin, 1984; Vahidnia et al., 2021). The individual repercussions for disregarded students are manifold, including poor classroom adaptability, disliking teachers, diminished interest in learning, and potential dropout (Martin, 1984; Davis and Lease, 2007; Mercer and DeRosier, 2010; Abadzi and Llambiri, 2011; Chiu et al., 2013). For the entire class, the prevalence of teachers’ pets engenders adverse effects, such as a compromised class atmosphere, reduced morale, and increased instances of conflicts (Babad, 1995; Somersalo et al., 2002; Chiu et al., 2013; Vahidnia et al., 2021). Although students designated as teacher’s pets are favored by instructors, they also endure negative consequences. They often face rejection by other students, becoming subjects of mockery, and at times even experiencing mistreatment and physical aggression from peers (Martin, 1984; Taylor, 1989; Babad, 1995; Chang et al., 2007; Lundqvist, 2019; Vahidnia et al., 2019). Furthermore, they might develop feelings of guilt or exhibit conceited and haughty behavior (Zhou and Wang, 2014).

Some educators, acknowledge and endorse the concept of “teachers’ pets” (Perlmutter, 2002), viewing these favored students as catalysts for enhancing teachers’ enthusiasm in the classroom. However, they are also aware that overtly displaying these preferences contradicts educational fairness, prompting them to adopt a more subtle approach (Tal and Babad, 1989). Yet, teachers frequently overestimate their ability to conceal these preferences while underestimating students’ awareness of the teacher’s pet phenomenon (TPP) (Trusz, 2017).Though teachers can consciously manage their verbal engagements with favored students, controlling facial expressions and body language poses a more considerable challenge. Unintentional non-verbal cues often reveal their distinct bond with these students, observable even by other classmates, including younger ones (Tal and Babad, 1989; Babad et al., 1991; Babad and Taylor, 1992). Moreover, biased or inexperienced teachers exhibit a more noticeable ‘leakage effect’ in showcasing favoritism, often purposefully obscuring the negative implications of their preferences (Babad et al., 1989; Trusz, 2017).

### 1.2 Characteristics of teacher’s pets

Tal and Babad (1989) identified several traits associated with students who tend to become teachers’ pets. These include active participation in teachers’ activities, superior intellectual attributes, and a tendency to obey and flatter teachers. Luttrell (1993) suggested that factors like race and skin color could also influence teachers’ preferences. In summary, students exhibiting these key characteristics are more likely to become teachers’ pets: those with commendable academic performance, not necessarily the highest achievers (Ritts et al., 1992; Babad, 1995); individuals with strong social communication skills, actively seeking to please teachers in daily interactions (Ritts et al., 1992; Babad, 1995; Trusz, 2017; Vahidnia et al., 2019); those demonstrating obedience and a peaceable personality, conforming to teachers’ directives (Babad, 1995; Trusz, 2017); students with attractive appearances (Ritts et al., 1992; Aydogan, 2008; Babad, 2009); and those hailing from families with higher social and economic status (Garrett and Crump, 1980; Luttrell, 1993; Aydogan, 2008; Hussain et al., 2019). However, at present, there is a lack of specialized empirical studies systematically investigating this issue.

The phenomenon of teachers’ pets remains pervasive in education globally, persisting from the past to the present day. Surprisingly, despite Babad’s work (Babad et al., 1989, 1991; Babad, 1995, 2009), few scholars have extensively studied this phenomenon. Consequently, the existing literature on teachers’ pets is limited, primarily focusing on primary, secondary, and higher education levels (Tal and Babad, 1990; Opoku-Amankwa, 2009; Chiu et al., 2013; Trusz, 2017; Lundqvist, 2019; Vahidnia et al., 2019, 2021), with relatively scarce exploration in preschool education (Taylor, 1989; Jiang, 2017; Wang and Wang, 2018). The study indicates that students in higher grades typically hold negative perceptions of teachers’ pets, exhibiting strong aversion. Conversely, younger students view teacher’s pets as role models, showcasing entirely different reactions (Brey and Pauker, 2019). This variance might stem from the limited self-judgment ability of young children, making them more susceptible to adult influence. Moreover, teachers’ pets often demonstrate commendable academic performance, a key factor contributing to differential treatment by teachers (Ritts et al., 1992; Babad, 1995). However, in preschool education, which is primarily play-based and lacks formal exams or academic benchmarks, the dynamics of the teachers’ pet phenomenon exhibit distinct characteristics, necessitating further refinement of study.

## 2 Methods

This study utilized an exploratory hybrid approach. The use of hybrid research contributes to achieving epistemological unity (Onweugbuzie, 2002). In hybrid research, quantitative and qualitative methods can collaboratively fulfill research purposes, and data and results can mutually validate, thereby enhancing the credibility and validity of the research (Johnson and Onwuegbuzie, 2004; Kelle, 2006; Walsh, 2014). Initially, qualitative study through interviews was employed to comprehend the types of children favored by kindergarten teachers. Interview data underwent coding, extracting, and classifying the categories of teachers’ pets. Subsequently, quantitative study ensued. Firstly, a questionnaire survey validated the model structure of teachers’ pet categories. Secondly, analytic hierarchy process, a combination of the entropy method, and a combination weight evaluation method based on game theory was employed to compute the weights of various indices for the types of teachers’ pets. Through these weight calculations, comparisons were conducted to ascertain which type of preschooler is more likely to garner attention from teachers and which indicators hold a greater sway over teachers’ favoritism toward preschoolers.

### 2.1 Qualitative study

The qualitative study collected data using a semi-structured interview method with questions centered on “What constitutes a teacher’s pet?”, “What characteristics do preferred children have?”, and “What are your views on the teacher’s pet phenomenon?”. The selection of interviewees followed two stages: The first employed purposive sampling, involving 13 kindergarten teachers selected based on teaching experience, educational background, geographical location, and kindergarten type to ensure diversity. The second stage utilized convenience sampling, interviewing two additional kindergarten teachers to verify data saturation and ensure comprehensiveness of content, exploring potential new categories of teachers’ pets. Basic information about the 15 respondents: 13 female teachers, 2 male teachers; 11 teachers from public kindergartens, 4 from private kindergartens; 5 with a master’s degree, 7 with a bachelor’s degree, and 3 with a junior college degree; 7 with teaching experience ≤3 years, 4 with experience ≤8 years, and 4 with experience >8 years. The total interview duration was 675 min. Each respondent was assigned a numerical ID from IT01 to IT15. All interviews were transcribed into text, totaling 12,800 words.

The processing and coding of interview materials relied on the bottom-up coding technique of grounded theory, facilitated by NVivo 11.0 software. The initial phase involved open coding to extract specific indices related to types of teacher-pet students. Subsequently, the process entailed categorizing, merging, and organizing these specific indices to construct a model representing teachers’ pets. To maintain objectivity and consistency throughout the data encoding process, three researchers collaborated on encoding the interview data.

### 2.2 Quantitative study

#### 2.2.1 Confirmatory factor analysis

Quantitative data for the study were gathered through a questionnaire survey that encompassed 7 types of teachers’ pets with 32 specific observation indices. The questionnaire utilized a Likert five-point scale, ranging from “very inconsistent” to “very consistent” for answer options. To ensure questionnaire content validity, 13 individuals (comprising 8 educators teaching preschool education in universities and 5 kindergarten principals) were invited by the author to evaluate its content validity. Assessment options included “irrelevant,” “weakly correlated,” “strongly correlated,” and “highly correlated.” Each question’s lowest I-CVI value was higher than 0.7, indicating good content validity for the questionnaire. Applying a random sampling method, a total of 545 questionnaires were collected. Samples with missing or extreme values were excluded, retaining 463 valid samples (Table 1). Mplus 8.0 was utilized to conduct confirmatory factor analysis on the samples, employing maximum likelihood estimation to assess the teacher’s pet model type.

**Table 1:** Basic information of samples.

Variables	Categories	Numbers N	Proportion%
Nature of kindergarten	Public kindergarten	288	62.2
	Private kindergarten	175	37.8
Location of kindergarten	City	368	79.5
	Village	95	20.5
Sex	Female	454	98.1
	Male	9	1.9
Degree	College and below	160	36.3
	Undergraduate	290	62.6
	Postgraduate	5	1.1
Kindergarten teacher certificate	With	365	78.2
	Without	98	21.2
Teaching experience	Teaching≤3 years	109	23.5
	3 years<Teaching≤8 years	136	29.4
	8 years<Teaching≤15 years	86	18.6
	15 years<Teaching	132	28.5

#### 2.2.2 Determine weights

Using survey data from 463 kindergarten teachers regarding the “types of teachers favoring students,” calculations were conducted. In the first step, the Analytic Hierarchy Process (AHP) was utilized to calculate the subjective weights of teacher-pet types and each indicator. AHP is considered an ideal subjective weight analysis method (Saaty, 1980; Lai et al., 2015). For this analysis, 17 professionals, including frontline kindergarten teachers with bachelor’s degrees or higher, were engaged. They underwent training to ensure accurate and meticulous completion. In the second step, the entropy method was employed to calculate the objective weights of teacher-pet types and each indicator. To eliminate the impact of measurement units, the indexes were standardized. In the final step, the combination weighting evaluation method in game theory was employed for comprehensive calculations. AHP is susceptible to the influence of experts’ knowledge, experience, and subjective biases, while the entropy method overlooks decision makers’ subjective judgments (Lai et al., 2015). Consequently, both methods have their limitations. In response, game theory methods were employed to amalgamate the weight sets derived from the Entropy Method and AHP, thereby establishing a novel evaluation method (Chen, 2003; Tables 2, 3).

**Table 2:** The relative importance of each index.

Relative importance	Specific meaning
1	Equal important
3	Weak Important
5	Important
7	Very important
9	Absolute Important
2, 4, 6, 8 between the above two judgments	

**Table 3:** The calculated and recommended random index values.

n	1	2	3	4	5	6	7	8
RI	0	0	0.58	0.9	1.12	1.24	1.32	1.41
n	9	10	11	12	13	14	15	
RI	1.46	1.49	1.52	1.54	1.56	1.58	1.59	

## 3 Results

### 3.1 Fits of teachers’ pets model

As revealed in the interviews, the typology of kindergarten teachers’ favored students is intricately captured through 32 distinct indices (refer to Table 4), further categorized into seven types: a child of good appearance (GA), a child possessing outstanding ability (OA), a child exhibiting good conduct (GC), a child with a proactive and enthusiastic character (PE), a child demonstrating obedience and carefree demeanor (OC), a child belonging to the vulnerable and concerned group (VC), and a child influenced by parental factors (PI). Specifically, GA denotes a teacher’s pet student with a commendable appearance, characterized by neat and beautiful attire. OA pertains to the advantageous abilities of teachers’ pets, including hands-on proficiency, interpersonal communication skills, self-care capabilities, language expression, and more. GC encompasses teachers and pet students exhibiting excellent qualities such as kindness, confidence, optimism, focus, and politeness. PE describes teachers’ pets adept at expressing affection to teachers and actively engaging with them. OC characterizes students who are meek, submissive, and adhere to teachers’ instructions. VC pertains to exceptional children facing physical disabilities, psychological challenges, and relatively disadvantaged family backgrounds. PI involves factors like favorable socio-economic conditions, elevated social status, and parents actively seeking to please teachers, leading to their children becoming teachers’ pets.

**Table 4:** Specific indexes of teacher’s pet types.

Dimension	Index	RP	M	SD
Good appearance (1–3)	(1) Dress cleanly, neatly, and beautifully	7	3.71	1.17
	(2) Behave cutely	8	3.60	1.17
	(3) Good-looking	6	3.44	1.15
Outstanding abilities (4–10)	(4) Strong hands-on operation ability	5	3.87	1.15
	(5) Strong timely reaction capacity	2	3.80	1.13
	(6) Strong interpersonal skills	5	3.74	1.14
	(7) Strong ability of living independence	6	3.89	1.12
	(8) Strong sports ability	4	3.63	1.10
	(9) Strong learning absorption ability	6	3.88	1.09
	(10) Strong speech language ability	2	3.87	1.10
Good conduct (11–16)	(11) Combat difficulties	2	3.95	1.08
	(12) Genuine and kind	9	4.00	1.12
	(13) Generous and confident	3	4.00	1.07
	(14) Optimistic	17	4.03	1.06
	(15) Focused	5	4.07	1.05
	(16) Good manner	3	4.10	1.07
Proactive and enthusiastic (17–19)	(17) Take the initiative to express love to teachers.	13	3.87	1.07
	(18) Proactively showcase oneself	3	3.77	1.04
	(19) Actively responding to teachers	2	3.98	1.05
Obedient and carefree (20–23)	(20) Lovely and quiet	10	3.84	1.09
	(21) Abide by rules and obey teacher’s instructions	6	3.91	1.08
	(22) Making teacher carefree	5	3.65	1.16
Vulnerable concerned (23–27)	(23) Physical disability	1	3.81	1.11
	(24) Psychological obstacles	2	3.67	1.11
	(25) Special family structure (for example, parents divorced, etc.)	2	3.72	1.04
	(26) From economically impoverished households	2	3.71	1.06
	(27) Neglected by parents in parenting	2	3.68	1.06
Parental influence (28–32)	(28) Parents maintain close communication with teachers	3	2.71	1.20
	(29) Parents cooperate and support teacher’s work	10	2.28	1.15
	(30) Parents with good interpersonal relationships	4	3.24	1.19
	(31) Parents with good socio-economic background	3	3.57	1.22
	(32) Parents giving gifts to please teachers	4	1.96	1.12

**Table 5:** Appropriateness of the internal structure of teacher’s pet types.

Types	RP	M	SD	ANVEQ	CR
GA	21	3.58	1.11	0.86	0.94
OA	30	3.81	1.04	0.83	0.97
GC	39	4.02	1.03	0.89	0.98
PE	18	3.87	1.00	0.84	0.94
OC	21	3.80	1.03	0.78	0.91
VC	9	3.72	0.97	0.75	0.93
PI	24	2.75	0.93	0.48	0.82

ANVEQ, Average Number of Variations Extraction Quantity; CR, Composite Reliability.

**Table 6:** Weight results of AHP.

Goal	Grade 1	Grade 2		Grade 1	Grade 2	
Teacher’s pet types	GA (0.073)	GA1	0.028	PE (0.174)	PE1	0.061
		GA2	0.028		PE2	0.022
		GA3	0.017		PE3	0.091
	OA (0.198)	OA1	0.015	OC (0.143)	OC1	0.043
		OA2	0.018		OC2	0.070
		OA3	0.037		OC3	0.030
		OA4	0.046	VC (0.145)	VC1	0.043
		OA5	0.012		VC2	0.034
		OA6	0.034		VC3	0.028
		OA7	0.037		VC4	0.016
	GC (0.173)	GC1	0.018		VC5	0.024
		GC2	0.029	PI (0.095)	PI1	0.021
		GC3	0.027		PI2	0.042
		GC4	0.031		PI3	0.014
		GC5	0.025		PI4	0.010
		GC6	0.042		PI5	0.007

**Table 7:** Weight result of entropy method.

Goal	Grade 1	Grade 2		Grade 1	Grade 2	
Teacher’s pet types	GA (0.135)	GA1	0.027	PE (0.088)	PE1	0.019
		GA2	0.029		PE2	0.019
		GA3	0.031		PE3	0.018
	OA (0.109)	OA1	0.027	OC (0.106)	OC1	0.021
		OA2	0.024		OC2	0.020
		OA3	0.025		OC3	0.027
		OA4	0.022	VC (0.101)	VC1	0.022
		OA5	0.024		VC2	0.024
		OA6	0.021		VC3	0.020
		OA7	0.021		VC4	0.022
	GC (0.0899)	GC1	0.019		VC5	0.021
		GC2	0.021	PI (0.370)	PI1	0.039
		GC3	0.022		PI2	0.032
		GC4	0.019		PI3	0.068
		GC5	0.017		PI4	0.104
		GC6	0.018		PI5	0.155

**Table 8:** Combination weight results based on game theory.

Goal	Grade 1	Grade 2		Grade 1	Grade 2	
Teacher’s pet types	GA (0.122)	GA1	0.028	PE (0.157)	PE1	0.037
		GA2	0.029		PE2	0.020
		GA3	0.025		PE3	0.048
	OA (0.114)	OA1	0.022	OC (0.148)	OC1	0.030
		OA2	0.021		OC2	0.041
		OA3	0.030		OC3	0.028
		OA4	0.032	VC (0.111)	VC1	0.031
		OA5	0.019		VC2	0.028
		OA6	0.026		VC3	0.023
		OA7	0.028		VC4	0.019
	GC (0.114)	GC1	0.019		VC5	0.022
		GC2	0.024	PI (0.243)	PI1	0.032
		GC3	0.024		PI2	0.036
		GC4	0.024		PI3	0.045
		GC5	0.021		PI4	0.065
		GC6	0.028		PI5	0.094

**Figure 1:**
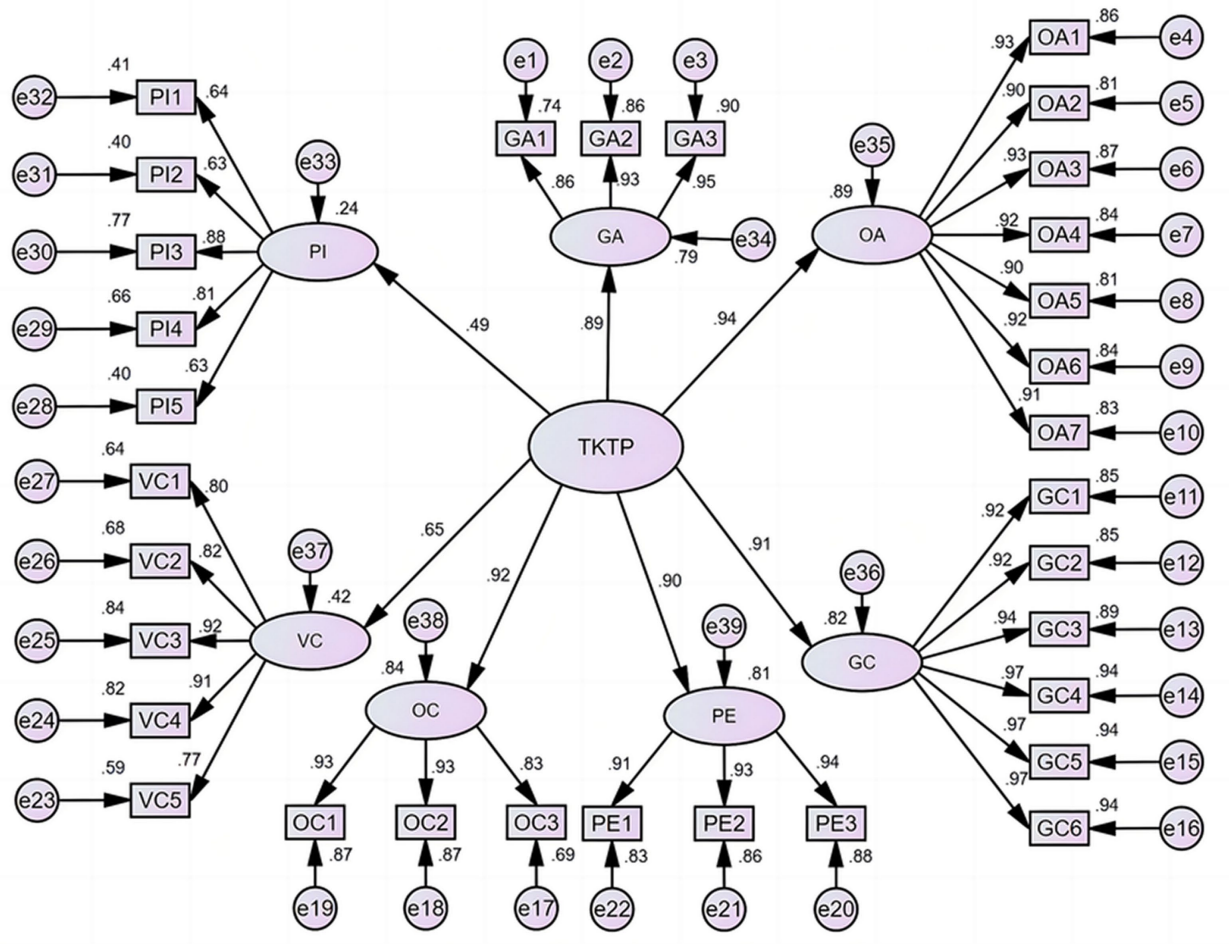
Teacher’s pet model.

The skewness coefficients of the observation indices (each question) range from 0.02 to 1.48, and the absolute values of the kurtosis coefficients range from 0.001 to 1.78. The error variances in the observation indices are not negative and reach a significant level. The decision value of the error variance falls between 7.68 and 14.09, both reaching a significant level above 0.001. The standard error of the parameter ranges from 0.006 to 0.063. As depicted in Figure 1, the factor load of each observation index for the seven types of teachers’ pets exceeds 0.6. Model fit indices are favorable: *X*^2^/df = 3.90, *p* < 0.001, CFI = 0.938, TLI = 0.930, RMSEA = 0.079, SRMR = 0.064. The overall suitability of this model is satisfactory, indicating the acceptability of the second-order model for kindergarten teacher-pet student typology.

The average variance extraction for the seven types exceeds 0.7, except for the parental influence type, which is 0.48. The combined reliability of all seven dimensions is above 0.8, meeting the criterion of greater than 0.6. The overall Cronbach’s Alpha coefficient of the questionnaire is 0.97, confirming the inherent structure of the model’s good fit (Table 5).

### 3.2 Weight results of teachers’ pets model

#### 3.2.1 Weight results of AHP

This study identified the types and specific indices of teachers’ pets through the aforementioned analysis. Among these, the types of teachers’ pets form the goal hierarchy, while the criteria hierarchy encompasses children with GA, OA, GC, PE, OC, and PI. The external case hierarchy includes 32 indexes. Seventeen experts participated in the evaluation, utilizing the data aggregation method of group decision-making experts for hierarchical analysis calculations. The software “yaahp” facilitated these computations. The average weight was selected for each expert. During the calculation process, the consistency test results of individual expert indices were inconclusive, necessitating the adoption of a method to modify the inconsistent judgment matrix. All CR (Consistency Ratio) values range from 0.000 to 0.0999, meeting the criterion of CR < 0.1. The final calculated weight results are presented in Table 6.

#### 3.2.2 Weight result of entropy method

**Figure 2:**
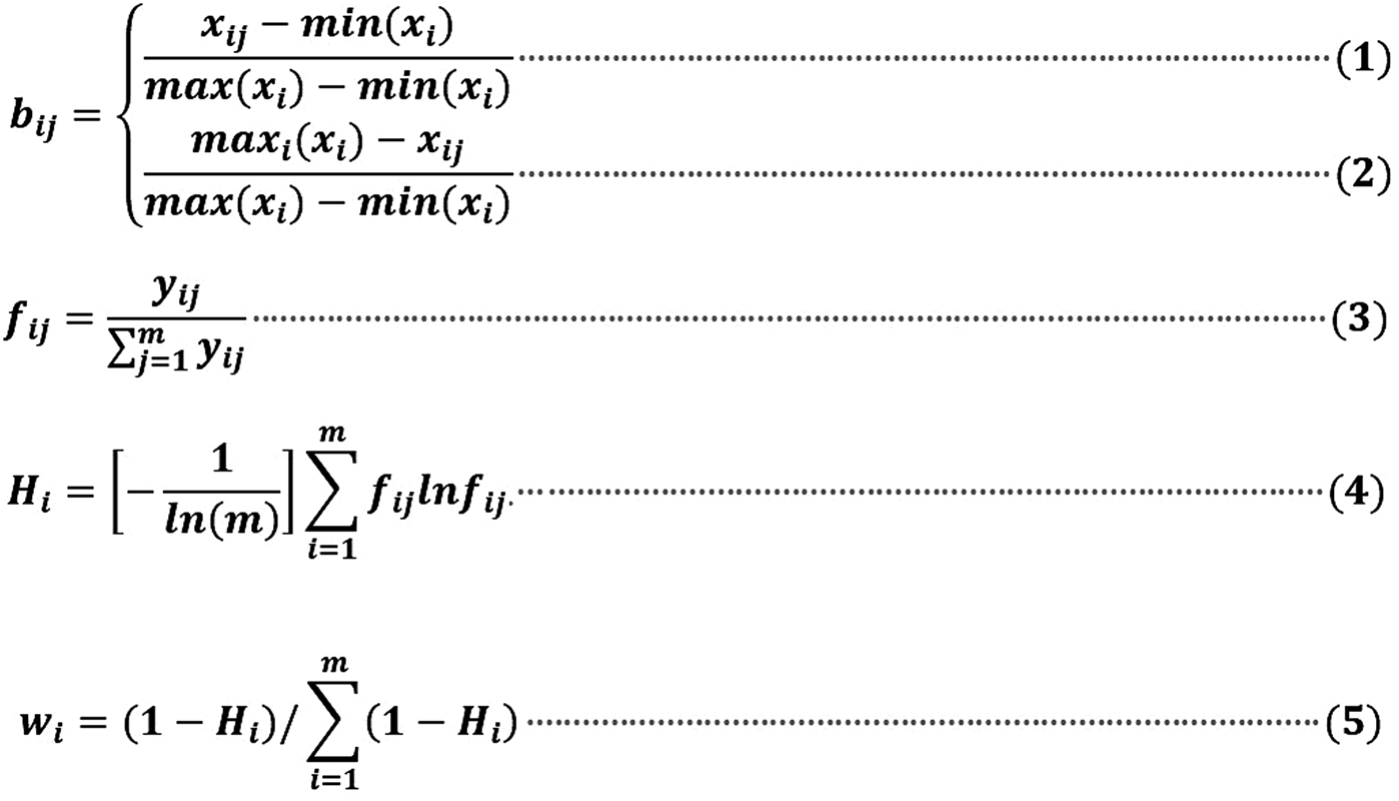
Weight result of entropy method.

This study utilized formulas (1) to (5), as shown in Figure 2, to calculate the weight results employing the Entropy Method. These results are detailed in Table 7.

#### 3.2.3 Combination weight results based on game theory

Utilizing a combination model of game theory with weights derived from the entropy method and AHP, the weight coefficients for the entropy method and AHP were calculated as α₁ = 0.584 and α₂ = 0.416, respectively. Table 8 displays the specific weights assigned to each index.

The final weight value in this study represents the amalgamated result based on game theory. As indicated in Table 7, the foremost three categories of teachers’ favored students include children influenced by parental factors (24.3%), proactive and enthusiastic children (15.7%), and obedient, carefree children (14.8%). Comparatively, within the spectrum of teachers’ preferred types, the vulnerable and concerned children (11.1%) hold the lowest weight, implying a lower likelihood of attention from teachers for this category of children.

In terms of specific indicators, the top three indices comprise parents who gift teachers to gain favor (9.4%), parents with robust socioeconomic backgrounds (6.5%), and children who actively engage with teachers (4.8%). These particular indices significantly influence kindergarten teachers’ preferences for children. On the contrary, the least-rated indices encompass children facing hardships (1.9%), children from economically disadvantaged households (1.9%), and children displaying exceptional athletic prowess (1.9%). This signifies that these three indicators exert the least influence on kindergarten teachers’ preferences for young children.

## 4 Discussion

### 4.1 A child of good appearance

The external appearance of young children can influence teachers’ preferences, consistent with previous study findings. When young children present themselves in a clean, neat, and attractive manner, they often attract the attention of teachers who are more willing to invest time and energy in accompanying them (Luttrell, 1993). The appearance of young children significantly impacts teachers’ preferences, as educators generally form more favorable impressions of children with esthetically pleasing looks (Ritts et al., 1992; Gu, 2013; Hussain et al., 2019). Conversely, children with less appealing appearances and untidy clothing are more likely to face rejection or discrimination from biased teachers (Feshbach, 1969; Felson, 1980). The lovability of young children is also a crucial factor influencing the extent of teacher preference (Tal and Babad, 1990). While a child’s handsome or beautiful appearance contributes to their cuteness, it’s essential to note that cuteness does not necessarily equate to a handsome and beautiful appearance. Even though some children may appear ordinary, they possess characteristics that attract teachers, such as a chubby figure and curly hair, leading them to become teachers’ pets (Luttrell, 1993; Babad, 2009).

### 4.2 A child possessing outstanding ability

Previous studies have established that students excelling academically are often favored by teachers and become their pets (Tal and Babad, 1989; Ritts et al., 1992; Mikami et al., 2012). Conversely, students with weaker academic performance are typically less preferred by teachers (Mikami et al., 2012). However, the kindergarten stage lacks formal exams and academic evaluations. Consequently, in contrast to prior studies, kindergarten teacher pets are not characterized by excellent academic performance. Academic prowess is a significant manifestation of students’ abilities, and kindergarten teacher pets exhibit outstanding abilities, such as hands-on operation, language expression, self-care, interpersonal communication, and athletic skills. Among these, exceptional interpersonal communication abilities have been consistently identified as a common trait among teacher pets (Ritts et al., 1992; Trang and Hansen, 2021). While previous studies have seldom highlighted being a teacher’s pet due to outstanding hands-on, self-care, sports, and language abilities, these qualities are notable among kindergarten teacher pets. Unlike pets in primary and secondary schools, where academic performance garners more attention, kindergarten teachers prefer pets with a broader spectrum of abilities. This inclination is shaped by the distinctive nature of kindergarten education.

### 4.3 A child exhibiting good conduct and a child with a proactive and enthusiastic character

During the kindergarten phase, young children are typically quite young and may not display significant utilitarian behaviors. While some children might actively express affection toward teachers, their primary motivation might not be to deliberately seek favor or gain benefits or privileges, although kindergarten teachers might provide certain preferential treatment to identified pet students. The preference of kindergarten teachers for specific young children reflects a proactive judgment on the part of the teacher. Certain children may be favored due to their exemplary qualities such as kindness, confidence, optimism, focus, and politeness. Studies have also shown that outstanding characteristics such as independence, confidence, and proactive interaction with teachers tend to earn favor from educators (Feshbach, 1969; Ritts et al., 1992; Babad, 2009).

Previous studies have suggested that the idea of teachers’ pets embodies a strong utilitarian nature, often marked by a proactive drive to please teachers and cultivate unique relationships with them, often in pursuit of unfair advantages or privileges, like higher grades (Silberman, 1969; Vahidnia et al., 2019). It has been observed that older students demonstrate a stronger tendency to seek favor with teachers, whereas younger students exhibit a weaker inclination in this regard. Studies indicate that among identified pet students, higher-grade levels tend to display more utilitarian behaviors (Tal and Babad, 1990; Vahidnia et al., 2021). Distinctions between senior and junior students also extend to their perceptions of teachers’ pets. In higher grades, identified pets may encounter aversion and rejection from other students (Lundqvist, 2019; Vahidnia et al., 2019). Conversely, younger students often view these pets as role models to emulate (Kuklinski and Weinstein, 2001; Mikami et al., 2012; Brey and Pauker, 2019).

### 4.4 A child demonstrating obedience and carefree demeanor

In the context of Chinese culture, obedience stands as a hallmark trait among kindergarten teacher pets. The educational emphasis in China often centers on students adhering to teachers’ instructions and collective regulations. Children displaying obedient characteristics tend to maintain a quiet, compliant demeanor, adhering to rules without causing disruptions, thereby potentially reducing teachers’ workload. Authoritative and burnt-out teachers often display a preference for young children exhibiting obedience (Babad, 1995; Chiu et al., 2013; Trusz, 2017). Similarly, in Western countries, teacher pets also demonstrate a strong trait of obedience. This attribute aids inexperienced teachers in effectively managing classroom discipline (Feshbach, 1969; Tal and Babad, 1989; Babad, 1995).

### 4.5 A child belonging to the vulnerable and concerned group

In prior studies, the notion of teachers’ pets has generally been portrayed as a passive and negative concept. Almost all studies have examined the phenomenon of teachers’ pets, contending that it results in educational inequality and has a negative impact on non-pet students (Babad, 1995; Vahidnia et al., 2021). However, this study contends that teachers’ pets are not an unequivocally negative phenomenon but also possess positive aspects. Among the categories of teacher pets, the group comprising vulnerable and concerned children represents a positive facet of teachers’ pets. Teachers actively direct their attention to students from vulnerable groups, encompassing those with physical disabilities, psychological disorders, family poverty, and exceptional children neglected by their parents, providing them with increased care and attention. Aligned with Rawls’ principle of justice, which posits that the least advantaged should receive the greatest benefits (Rawls, 2009), it is unfortunate that, among the various types of teacher pets, children identified as vulnerable and concerned (11.1%) carry the lowest weight compared to other categories. The inclination of vulnerable and concerned children toward teachers signifies a manifestation of educational fairness, contributing to the physical and mental well-being of these vulnerable children. This positive aspect should be further encouraged and promoted.

### 4.6 A child influenced by parental factors

School is a battleground for identity struggles, and parents play a crucial role in determining whether students can become teachers’ pets (Luttrell, 1993). The findings of this study reveal that, in comparison to other types of teachers’ pets, children influenced by parental factors (24.3%) hold the highest weight. This suggests that, within the cultural context of China, parents exert the most significant influence in determining whether children receive preferential treatment from teachers. Existing studies predominantly focus on the socioeconomic status of students’ parents. Children of parents with elevated social status, economic prosperity, or political influence often receive greater favoritism from teachers (Babad et al., 1989; Wang, 2002; Gu, 2013). Such children are more likely to be preferred by teachers if their parents are relatives, friends, or hold positions of authority within the kindergarten’s management or among colleagues (Hussain et al., 2019). This type of preference exhibits particularities, with teachers sometimes actively providing favoritism and other times doing so passively. In this category, even if some young children are not personally favored by teachers, they may receive special preferential treatment due to their parents’ influence. Some parents actively seek to please teachers by offering gifts or money, aiming to secure special attention for their children from teachers. Although these children are more likely to receive favor from teachers, it’s crucial to highlight that giving gifts to teachers, considered as bribery, is explicitly prohibited in China due to ethical standards for educators. Teachers who accept gifts from parents might encounter repercussions. Consequently, these exchanges frequently take place discreetly and have proven difficult to entirely eradicate.

## 5 Conclusion, limitations, and future prospects

The types of kindergarten teachers’ pets are specifically delineated by 32 indices, categorized into 7 types: a child of good appearance (GA); a child having outstanding ability (OA); a child having good conduct (GC); a child with proactive and enthusiastic character (PE); a child of obedient and carefree (OC); a child belong to the vulnerable concerned (VC); a child with parental influence (PI). The model structure encompassing these 7 types of teacher’ pets is considered reasonable based on confirmatory factor analysis and weight determination. Some types align with existing studies (GA, OA, GC, PE, and OC), while others present new characteristics (VC and PI).

This study has two limitations. Firstly, it is situated within the cultural context of China, introducing a degree of specificity to the results. Therefore, the findings may not be entirely generalizable to other countries. Secondly, the study primarily analyzes preference types among kindergarten teachers without further investigating the impact of teacher favoritism. This issue deserves further exploration in subsequent studies.

## Data availability statement

The raw data supporting the conclusions of this article will be made available by the authors, without undue reservation.

## Ethics statement

The studies involving humans were approved by the Ethics Committee of Nantong University/Nantong University. The studies were conducted in accordance with the local legislation and institutional requirements. The participants provided their written informed consent to participate in this study.

## Author contributions

SS: Conceptualization, Methodology, Project administration, Supervision, Writing – original draft. HZ: Conceptualization, Project administration, Validation, Writing – original draft. ZZ: Writing – review & editing. JS: Data curation, Formal analysis, Writing – review & editing. XQ: Formal analysis, Investigation, Writing – review & editing. SL: Writing – review & editing.
